# Studies on Dissipations and Residues of Indoxacarb under Different Field and Environmental Conditions

**DOI:** 10.1155/2020/8874759

**Published:** 2020-11-04

**Authors:** Ying-Hong Li, Xiang-Yun Wang, Wei Hua, Hu Zhang

**Affiliations:** ^1^Zhejiang Institute for Food and Drug Control, NMPA Key Laboratory for Testing and Warning of Pharmceutical Microbiology, Hangzhou 310052, China; ^2^Agricultural Ministry Key Laboratory for Pesticide Residue Detection, Zhejiang Province Key Laboratory for Food Safety, Institute of Quality and Standard for Agro-products, Zhejiang Academy of Agricultural Sciences, Hangzhou 310021, China; ^3^Sipcam Crop Science (Wuxi) Co Ltd., Wuxi, China

## Abstract

Indoxacarb is a broad-spectrum insecticide and widely used in agriculture. The dissipations and residues of indoxacarb were researched at three different field sites in Beijing, Hunan, and Zhejiang provinces in China. Analytical methods for determining the residue of indoxacarb in paddy water, paddy soil, rice straw, rice hull, and brown rice were described. Indoxacarb residues were extracted from samples, cleaned up by solid phase extraction, and determined by high-performance liquid chromatography coupled with tandem mass spectrometry in the selected ion monitoring mode. The recoveries in paddy water, paddy soil, rice straw, rice hull, and brown rice matrices at three spiking levels ranged from 79.7% to 98.3%, respectively. The field and environmental conditions would affect the dissipations and residues of indoxacarb. The time to dissipate 50% of indoxacarb in paddy water, paddy soil, and rice straw was less than 9 days. The terminal residues obtained from Beijing at preharvest interval of 14 and 21 days were higher than the maximum limit of European Union. Therefore, a dosage of 24 g a. i. ha^−1^ at 28 days preharvest interval with 3 spraying times was recommended. Such accumulation of measured data is necessary to provide guidance for the proper and safe use of this pesticide.

## 1. Introduction

With the continuous growth of global population, people's demand for food is also increasing. In order to meet this demand, farmers use pesticides to improve agricultural productions and protect crops from damage in the agricultural process [1]. Pesticides are defined as substances or chemicals mainly used to increase crop yield in agriculture, horticulture, forestry, and public land, which can shorten the growth period of agricultural products, increase the productions, and reduce the various diseases of crops [2]. However, in most cases, pesticides can penetrate into plants and transfer to edible parts of crops. When farmers use pesticides improperly in the agricultural process, pesticide residues in products may exceed the maximum residue limit (MRL) specified in the current standards, thus causing a potential risk to the health and safety of consumers [3–6].

Residues of pesticide in agricultural environments such as water, soil, and crops are major pollutions in the agriculture [7]. It is essentially important to study environmental fates and exposure models to integrate information on chemicals, their partitioning, and degradation behaviors [8, 9]. The main path for pesticide degradation includes microbiological deterioration, hydrolysis, and photolysis [10, 11]. Moreover, the effects such as soil type, temperature, and moisture on pesticide degradations are analyzed and reported [12, 13]. These researches can help to provide data for the further remediation of contaminated environments by pesticide.

Indoxacarb{methyl-7-chloro-2, 5-dihydro-2-[[(methoxycarbonyl)[4-(trifluoromethoxy) phenyl] amino] carbonyl] indeno [1, 2-e] [1, 3, 4]-oxadiazine-4a (3H)-carboxylate} is a popular insecticide, which is the first commercialized pyrazoline-type sodium-channel blocker [14]. Indoxacarb is a broad-spectrum insecticide effective against lepidopteran pests and selected sucking pests, such as flea hoppers and tarnished bugs. It has a novel mode of action that blocks the movement of sodium ions into certain nerve cell ion channels, resulting in paralysis and death of pests [15]. Now, it has been registered in many countries and used on crops, vegetables, and fruits. However, chronical and continuous application of indoxacarb also results in negative environmental consequences. More and more attentions have been paid to the toxicity of indoxacarb [16]. It is revealed that sublethal concentrations of indoxacarb would cause metabolism alteration, hydromineral imbalance, and gill and kidney damages in common carp [17]. The European Food Safety Authority (EFSA) also has demonstrated the initial risk assessments of indoxacarb [18]. Previous studies have investigated the dissipation behavior of indoxacarb in vegetables, fruits, and soil [19]. Few studies have investigated the dissipations and residues of indoxacarb under different field and environmental conditions [20, 21].

Rice is the most widely consumed food for a high proportion of global population, especially in Asia. It is necessary to confirm whether these chemicals are dissipated from the environment soon after their mission is accomplished [22–25]. According to the growing environment of rice plants, the dissipation of pesticides is definitely a complicated process [26]. It may relate to the entire rice growth season, rather than a certain stage [27, 28]. The present study is performed in open rice fields in Beijing, Hunan, and Zhejiang provinces. This work aims to establish a simple, fast, and efficient analytical method to detect and evaluate the dissipations and residues of indoxacarb in paddy water, paddy soil, rice straw, rice hull, and brown rice under different field and environmental conditions. This would help to provide basic information for developing regulations to guard safe use of indoxacarb in pest management strategies in rice fields and to protect public health.

## 2. Materials and Methods

### 2.1. Materials and Chemicals

Acetonitrile and methanol with guaranteed reagent grade were purchased from Merck (Darmstadt, Germany). Formic acid was supplied by Tedia company (Fairfield, USA), and ammonium acetate was purchased from Sigma company (Santa clara, USA). Water (18.2 M*ω* · cm) used for reagent, and sample preparation was from a Barnstead Nanopure system (Thermo Scientific, USA). All other chemicals were of analytical reagent grade and obtained from commercial sources. The analytical standard of indoxacarb (purity 99.5%) was purchased from Dr. Ehrenstorfer GmbH (Augsburg, Germany). Stock solutions were prepared by dissolving indoxacarb in acetonitrile. Working standards at lower concentrations were prepared by serial dilution of the stock standards and kept at 4°C.

### 2.2. Field experiments

Field experiments were performed in Beijing, Hunan, and Zhejiang provinces according to *the guideline for pesticide residue field experiments* issued by the Institute of the Control of Agrochemicals, Ministry and Agriculture, the People's Republic of China. These three provinces are located in different monsoonal climates and thus reflect various climatic and environmental conditions in China. The designs of the dissipations and residues field experiments are shown in Table 1. There were 5 treatments with 3 replicates and 1 control. Each experiment plot was 30 m^2^. No pesticide was used during the entire period of rice growth in the control plot. A buffer area of 30 m^2^ was used to separate the plots of different treatments.

To investigate the dissipation dynamics of indoxacarb in paddy water, paddy soil, and rice straw, indoxacarb 8% SC was sprayed at an active ingredient dose of 36 g a. i. ha^−1^ (1.5-fold higher of the recommended high dosage) on the rice crops. Water samples were collected randomly using a 500 mL cup and then mixed in a barrel. Soil samples were collected randomly from each plot using a soil auger to a depth of 10 cm from the surface. Plant samples with roots were collected and washed. Representative paddy water, paddy soil, and rice straw samples were collected randomly at several time points in each plot at 2 h (calculated as the original concentration) and 1, 3, 5, 7, 14, 21, and 28 days after spraying.

For the residue experiment, the indoxacarb 8% SC solution was applied at a low dosage of 24 g a. i. ha^−1^ (recommended high dosage) and a high dosage of 36 g a. i. ha^−1^ (1.5-fold higher of the recommended high dosage) for three and four applications with preharvest intervals 14, 21, and 28 days, respectively. The paddy soil, rice straw, rice hull, and brown rice were sampled at preharvest intervals of 14, 21, and 28 days after the last pesticide application for residue experiments. Rice was air-dried at room temperature and shelled into rice hull and brown rice. Brown rice was further grated to powder. All samples were placed in a deep freezer at −18°C and analyzed within 2 months.

### 2.3. Sample Preparations and Extraction Procedures

#### 2.3.1. Paddy Water

Hundred milliliters paddy water sample after filtration was transferred to the solid-phase extraction (SPE) column (C_18_, 1g, 6 mL), which was conditioned with methanol (5 mL) and water (5 mL). The cartridge was washed with water (5 mL). The residual water was removed under vacuum, and analytes were eluted into glass tubes by the addition of methanol (10 mL) under gravity flow. Extracts were evaporated to dryness under a nitrogen stream in a water bath at 40°C, the residue was redissolved in methanol (5 mL), and the tube was vortex-mixed for 10 s and after filtration through a 0.2 *μ*m syringe filter with nylon membrane and then transferred to a screw-cap vial.

#### 2.3.2. Paddy Soil

Acetonitrile (80 mL) and water (20 mL) were added to the 250 mL triangular flask containing 40 g soil sample. Vacuum filtration was done after being shaken vigorously for 1 h and then the filtrate was transferred to the measuring cylinder with 15 g of sodium chloride. The measuring cylinder was tightly sealed and shaken for 2 min and then placed for 40 min. The supernatant solution (20 mL) was transferred to a glass tube and evaporated under a nitrogen stream in a water bath at 40°C. The residue was dissolved in methanol and dichloromethane (5 : 95, v/v, 2 mL). A cartridge (NH_2_, 500 mg, 6 mL) was conditioned with methanol and dichloromethane (5 : 95, v/v, 5 mL), and then the above liquid was loaded on the cartridge. The cartridge was washed with the other methanol and dichloromethane (5 : 95, v/v, 8 mL) twice. All the above liquids were collected into glass tube and evaporated to dryness under a nitrogen stream in a water bath at 40°C. The residue was redissolved in methanol and water (1 : 1, v/v, 5 mL) and vortex-mixed for 10 s. The final solution was filtered through a 0.2 *μ*m syringe filter with nylon membrane before HPLC-MS/MS analysis.

#### 2.3.3. Rice Straw, Rice Hull, and Brown Rice

Acetonitrile (80 mL) and water (20 mL) were added to the 250 mL triangular flask containing 10 g samples (rice straw, rice hull, and brown rice). Sample preparation and extraction procedure were processed as described in Section 2.3.2.

### 2.4. HPLC-MS/MS Analysis

#### 2.4.1. Assay Method

HPLC-MS/MS detection was used for residue analysis by the TSQ Quantum Discovery mass spectrometer system (Thermo Fisher Scientific, USA) equipped with an electrospray interface. Thermo Fisher Xcalibur 2.0.7 software was used to control the instrument and collect and analyze data.

#### 2.4.2. HPLC Conditions

Separation was carried out on a column Luna C_18_ (150 mm × 2.0 mm, 5 *μ*m) supplied by Phenomenex (Torrance, USA). The mobile phase consisted of 90% (v/v) methanol and 10% (v/v) combined with 0.1% formic acid solution. The flow rate was set at 0.3 mL · min^−1^. Column oven temperature was set at 25°C, and the injection volume was 5 *μ*L.

#### 2.4.3. MS/MS Conditions

Electrospray in positive mode was used, and the spray voltage was 4.0 kV. The capillary temperature was 350°C. Aux auxiliary gas and sheath gas were normal nitrogen. Collision gas was high pure argon with pressure at 0.2 Pa in collision cell. The first mass transition was used for quantification, while the second mass transition was used for confirmation of the residues.  Table 2 shows the MS/MS transitions selected for quantification and confirmation together with the optimized parameters for indoxacarb. The retention time of indoxacarb was about 1.72 min.

### 2.5. Dissipation and Residue Assessment

The dissipations and residues assessment of indoxacarb were figured out by the HPLC −MS/MS method. The exponential function *C* = *C*_0_e^−kt^ was used as mathematical expressive model for pesticide dissipations, where *t* denotes time after pesticide application. Mathematical curve fitting was identified by computer-associated calculation on the basis of measured data.

## 3. Results and Discussion

### 3.1. Optimization of Sample Pretreatment

Acetonitrile, ethyl acetate, and methanol are usually chosen as the extraction solvent for pesticide analysis [29, 30]. In this study, acetonitrile and methanol were selected as the extraction solvents. SPE is a technique designed for rapid sample preparation and purification before chromatographic analysis [31, 32]. In this study, C_18_ and NH_2_ cartridges were employed for clean-up of complex matrices. The pretreatments consisted of three steps. Firstly, extraction with suitable solvents, then clean-up by SPE technique, and finally comprised concentration, reconstitution, and filtration.

### 3.2. Validation of Analysis Method

Matrix effect is a significant drawback in HPLC -MS/MS quantitative analysis [33, 34]. Thus, matrix effect had been investigated by comparing the detector responses from standard solutions in mobile phase with those from different matrices. The result showed that there were no significant matrix effects for indoxacarb in paddy water, paddy soil, rice straw, rice hull, and brown rice. Standard solution (0.01–1.0 mg/L) was chosen to calibrate for samples in this study. The linear equation was *y* = 18702771 *x* + 185338, with correlation coefficients (*r*^2^) 1.000. Limit of detection (LOD) for indoxacarb was calculated as the sample concentration (S/N ratio of 3), and the limit of quantification (LOQ) was defined as S/N ratio of 10. The LODs and LOQs are listed in Table 3.

A spiked recovery method was applied, in which standard solution was spiked in paddy water, paddy soil, rice straw, rice hull, and brown rice matrices at three concentration levels. A total of five replicate measurements were performed for each concentration level. Table 3 also lists the recoveries at three spiking levels of different matrices. Average recoveries of indoxacarb ranged from 79.7% to 98.3% in paddy water, paddy soil, rice straw, rice hull, and brown rice samples. The precision of the method, in terms of the relative standard deviations (RSD), ranged from 2.2% to 9.3%, respectively. The results illustrated that the methods were reliable and sensitive to determine indoxacarb in paddy water, paddy soil, rice straw, rice hull, and brown rice.

### 3.3. Results of Dissipation Data

**Figure 1:**
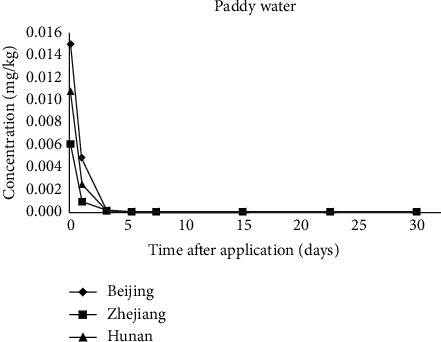
Dissipations of indoxacarb in paddy water in Beijing, Hunan, and Zhejiang.

Mathematical analysis for curve fitting was carried out on the basis of detected indoxacarb concentration which was varied over time.  Figure 1 is the dissipation curves of indoxacarb in paddy water. The dissipation dynamics of indoxacarb in three regions and provinces could be described as the following first-order kinetic equations: *C* = 0.0166*e*^−1.3563*t*^ (Beijing), *C* = 0.0060*e*^−0.6084*t*^ (Hunan), and *C* = 0.0048*e*^−1.0248*t*^ (Zhejiang), respectively. The time to dissipate 50% (DT_50_) of indoxacarb in paddy water calculated from the regression equation was 0.5, 1.1, and 0.7 days in Beijing, Hunan, and Zhejiang, respectively. The data indicated that indoxacarb dissipated rapidly in paddy water at all three sites and that the dissipation of indoxacarb in paddy water was not affected by the weather obviously.

**Figure 2:**
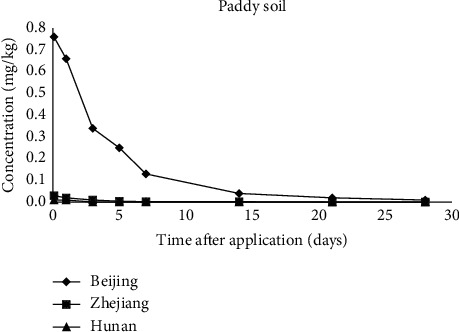
Dissipations of indoxacarb in paddy soil in Beijing, Hunan, and Zhejiang.

Figure 2 was the dissipation curves of indoxacarb in paddy soil. A gradual and continuous dissipation of indoxacarb was observed according to first-order kinetics. The dissipation dynamics of indoxacarb in soil could be described as the following equations: *C* = 0.5466*e*^−0.1528*t*^ (Beijing), *C* = 0.0053*e*^−0.0824*t*^ (Hunan), and *C* = 0.0181*e*^−0.2347*t*^ (Zhejiang), respectively. The DT_50_ for indoxacarb in soil was 4.5, 8.4, and 3.0 days in Beijing, Hunan, and Zhejiang, respectively.  Table 4 lists the environmental conditions in Beijing, Hunan, and Zhejiang. Soil of Beijing and Zhejiang experimental locations contains clay loam, with the organic matter content 1.4% and 1.08%, respectively. While Hunan experimental location is Doras loam with the organic matter content 11.00%. The organic matter of Hunan soil sample is about tenfold of that of the Beijing and Zhejiang soil.

The ability of soil to remove chemical contamination is primarily dependent on the presence of a microbial community [35]. Hydrophobic pesticides can be strongly sorbed by the organic matter of the soil, with decreased bioavailability of the compound to be degraded by soil microorganisms [36]. Furthermore, it is mentioned that soil organic matter, indigenous microorganisms, and contact time reduce desorption [37]. These may explain the lower degradation rates observed in soil of Hunan. In addition, the faster degradation rates of indoxacarb in soil of Zhejiang than that of Beijing may due to the annual average rainfall, which is 730.7 mm and 1959.5 mm in Beijing and Zhejiang (Table 4), respectively. Bacterial band richness of soil microorganisms is higher in normal rain regimes than drought regimes [38]. Significantly greater quantities of degradation products can be measured in the water-saturated surface soil compared to the unsaturated soil [39]. Thus, soil of Zhejiang is favourable to the degradation of indoxacarb.

**Figure 3:**
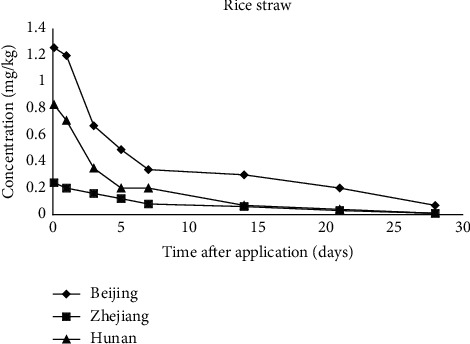
Dissipations of indoxacarb in rice straw in Beijing, Hunan, and Zhejiang.

Figure 3 is the dissipation curves of indoxacarb in rice straw. The first-order kinetics equations were *C* = 0.9896*e*^−0.0894*t*^ (Beijing), *C* = 0.5861*e*^−0.1265*t*^ (Hunan), and *C* = 0.217*e*^−0.0995*t*^ (Zhejiang), respectively. The DT_50_ of indoxacarb in rice straw was 7.8, 5.5, and 7.0 days in Beijing, Hunan, and Zhejiang, respectively. The dissipation of pesticide in rice straw is related with the metabolism ability of rice straw. The main factors which influence the growth and the development of rice crops are temperature, soil conditions, humidity, rainfall, etc. The difference of DT_50_ of indoxacarb in rice straw may result from the actual different parameters listed in Table 4.

In conclusion, indoxacarb dissipated quickly in paddy water, paddy soil, and rice straw in eastern, north, and central of China. The DT_50_ values of all analyzed matrices are summarized in Table 5. The DT_50_ for indoxacarb in three matrices are lower than 9 days. Based on Figures 1–3, it is clear that pesticide contamination occurs via a number of routes such as arable soils, soil water, and plants [40]. As shown in Figures 1–3, initial concentrations of indoxacarb at time 0 in analyzed matrices from different province are different from each other. It is generally known that pesticides are not only metabolized in organisms but also dissipated by many factors in the environment. The original deposition amount of pesticides on matrices varies with the types of matrices.

### 3.4. Results of Residue Assessment

Indoxacarb was detectable in paddy soil, rice straw, rice hull, and brown rice samples harvested after intervals of 14, 21, and 28 days (Table 6). The residues in paddy soil samples harvested after intervals of 14, 21, and 28 days in the three experimental locations ranged from <LOQ to 0.022 mg/kg. The residues in rice straw samples were between 0.029 mg/kg and 2.240 mg/kg, and in rice hull, it ranged from <LOQ to 2.131 mg/kg. At the same time, the residues of indoxacarb in rice straw and rice hull were higher than that of paddy soil. Indoxacarb was not detected in paddy soil samples harvested after intervals of 28 days in Zhejiang. All the residues in rice straw and rice hull were lower than 1.970 mg/kg after intervals of 28 days.

The residues of indoxacarb in brown rice in Beijing samples were more than 0.02 mg/kg at 21 days after spraying at the tested dosages. The total concentrations of indoxacarb residues in rice straw and rice hull were as in order of Beijing > Zhejiang > Hunan. The higher organic matter content of soil in Hunan (11%) may cause rice plants with stronger vigor of growth and roots than that of Zhejiang (1.08%), which results in stronger metabolism ability and lower indoxacarb residues in rice straw and rice hull. At the same time, comparisons of the indoxacarb residues in brown rice reveal that Beijing is with the highest residues while Hunan with the smallest residues. The results may be related to the locate weather. The annual average temperature is 12.9°C, 17.6°C and 17.0°C in Beijing, Hunan, and Zhejiang, respectively (Table 4). Indoxacarb may dissipate more quickly under higher temperature. In addition, the smallest annual average relative humidity in Beijing (51%, Table 4) may cause the lower microbial decomposition ability in Beijing brown rice samples [40]. The maximum limit of indoxacarb in brown rice in European Union is 0.02 mg/kg.

Hence, a safe preharvest interval of 28 days is suggested before harvesting of rice. The present finding suggests that the indoxacarb 8% SC could be used in rice field safely with the recommended dosage (24 g a. i. hm^−1^) at 28 days preharvest interval with 3 spraying times.

## 4. Conclusion

The results of this study indicate a practical approach to study dissipations and residues of indoxacarb in paddy water, paddy soil, rice straw, rice hull, and brown rice under different field and environmental conditions by the HPLC-MS/MS method. The method is validated reliable and sensitive to determine indoxacarb in different matrices.

The DT_50_ for indoxacarb in paddy water, paddy soil, and rice straw is less than 9 days. The terminal residue of indoxacarb in Beijing is higher than the maximum limit of EU at preharvest intervals of 14 and 21 days with the recommended dosage. Therefore, a dosage of 24 g a. i. hm^−1^ at 28 days preharvest interval with 3 spraying times is recommended. It provides data for the Chinese government to establish the maximum residue level of indoxacarb in rice and supports guidance on the proper and safe use of this pesticide.

## Figures and Tables

**Table 1:** Design of field experiments for the residue and dissipation researches.

Treatments		Dosage of application (g a.i ha^−1^)	Number of applications	Experiments	Time of sampling (days)
Serial number	Area (m^2^)				
1, 2, 3	30 × 3	24	3	Residues in paddy soil, rice straw, rice hull, and brown rice	14, 21, and 28
4, 5, 6	30 × 3	24	4		
7, 8, 9	30 × 3	36	3		
10, 11, 12	30 × 3	36	4		

13, 14, 15	30 × 3	36	1	Dissipations in paddy water, paddy soil, and rice straw	2 h, 1, 3, 5, 7, 14, 21, and 28

16	30 × 3	0	0	Control	2 h, 1, 3, 5, 7, 14, 21, and 28

**Table 2:** SRM conditions for indoxacarb.

Compound	Parent mass (*m*/*z*)	Product mass (*m*/*z*)	Collision energy (V)
Indoxacarb	528	249	20
	528	203^*∗*^	17

^*∗*^Quantitative ion

**Table 3:** LODs, LOQs, and average recoveries of indoxacarb in five matrices.

Matrices	LODs (*μ*g/kg)	LOQs (*μ*g/kg)	Average recoveries (*n* = 5)		
			Added level (mg/kg)	Mean ± SD (%)	RSD (%)
Paddy water	0.015	0.050	0.01	79.7 ± 7.0	8.8
			0.1	86.7 ± 7.3	8.4
			1.0	92.8 ± 4.4	4.7

Paddy soil	0.30	1.0	0.01	88.9 ± 6.0	6.8
			0.1	94.3 ± 4.4	4.7
			1.0	92.1 ± 2.5	2.7

Rice straw	0.67	2.0	0.01	83.0 ± 2.2	2.6
			0.1	96.5 ± 3.3	3.4
			1.0	95.5 ± 8.9	9.3

Rice hull	1.3	4.0	0.01	88.9 ± 2.0	2.2
			0.1	92.8 ± 4.3	4.6
			1.0	96.3 ± 3.8	3.9

Brown rice	0.67	2.0	0.01	98.3 ± 6.3	6.4
			0.1	94.8 ± 3.9	4.1
			1.0	95.2 ± 3.0	3.1

**Table 4:** The field and environmental conditions in Beijing, Hunan, and Zhejiang.

Field and environmental conditions	Beijing	Hunan	Zhejiang
Climate type	Seasonal temperate semihumid monsoon climate	Humid continental monsoon climate	Subtropical monsoon climate

Annual average temperature (°C)	12.9	17.6	17.0

Annual average relative humidity (%)	51	81	82

Annual average rainfall (mm)	730.7	1730.0	1959.5

Soil type	Clay loam	Doras loam	Clay loam

Organic matter content of soil (%)	1.40	11.00	1.08

pH of soil	7.9	5.5	6.0

**Table 5:** The DT_50_ values of indoxacarb in different matrices in Beijing, Hunan, and Zhejiang.

Matrices	DT_50_ values (days)		
	Beijing	Hunan	Zhejiang
Paddy water	0.5	1.1	0.7
Paddy soil	4.5	8.4	3.0
Rice straw	7.8	5.5	7.0

**Table 6:** Ultimate residues of indoxacarb in paddy soil, rice straw, rice hull, and brown rice (mg/kg).

Region	Dosage (g a. i. ha^−1^)	Spraying time	Preharvest interval (days)	Residues of indoxacarb (mg/kg)			
				Paddy soil	Rice straw	Rice hull	Brown rice
Beijing	24	3	14	0.004	0.950	1.211	0.046
			21	0.002	1.310	0.831	0.026
			28	0.002	0.810	0.911	0.019
		4	14	0.006	1.320	1.880	0.057
			21	0.002	1.490	0.920	0.099
			28	0.003	1.300	0.990	0.027
	36	3	14	0.009	1.560	1.960	0.065
			21	0.004	1.730	1.130	0.065
			28	0.005	1.650	1.081	0.032
		4	14	0.010	2.240	2.131	0.071
			21	0.011	2.180	1.431	0.072
			28	0.006	1.970	1.191	0.091

Hunan	24	3	14	0.001	0.172	0.016	<LOQ
			21	0.003	0.082	0.018	<LOQ
			28	0.002	0.042	0.010	<LOQ
		4	14	0.005	0.203	0.024	<LOQ
			21	0.002	0.181	0.022	<LOQ
			28	0.002	0.172	0.016	<LOQ
	36	3	14	0.009	0.291	0.034	<LOQ
			21	0.006	0.341	0.026	<LOQ
			28	0.008	0.231	0.021	<LOQ
		4	14	0.010	0.470	0.045	<LOQ
			21	0.011	0.392	0.029	<LOQ
			28	0.011	0.351	0.023	<LOQ

Zhejiang	24	3	14	<LOQ	0.151	0.611	0.019
			21	<LOQ	0.161	0.262	0.016
			28	<LOQ	0.029	0.093	<LOQ
		4	14	0.001	0.211	0.831	0.024
			21	<LOQ	0.219	0.601	0.020
			28	<LOQ	0.069	0.521	0.008
	36	3	14	0.004	0.281	1.372	0.029
			21	0.003	0.221	0.961	0.025
			28	0.001	0.142	0.642	0.014
		4	14	0.022	0.343	1.491	0.047
			21	0.004	0.252	1.321	0.034
			28	0.002	0.241	1.431	0.020

## Data Availability

The data used to support the study are included within the article.
